# Activation and Regulation of NLRP3 by Sterile and Infectious Insults

**DOI:** 10.3389/fimmu.2022.896353

**Published:** 2022-05-12

**Authors:** Srijon K. Banerjee, Ayan Chatterjee, Shamba Gupta, Abhinit Nagar

**Affiliations:** ^1^ Department of Microbiology and Molecular Genetics, School of Medicine, University of Pittsburgh, Pittsburgh, PA, United States; ^2^ Department of Microbiology and Molecular Genetics, McGovern Medical School, University of Texas Health Science Center at Houston, Houston, TX, United States; ^3^ Division of Genetics, Wadsworth Center, New York State Department of Health, Albany, NY, United States; ^4^ Flow Cytometry, Luminex Corporation, Austin, TX, United States

**Keywords:** NLRP3, Inflammasome, Microbes, Sterile Inflammation, extracellular vesicles, post-translational modification

## Abstract

Nod-Like Receptor (NLR) is the largest family of Pathogen Recognition Receptors (PRRs) that patrols the cytosolic environment. NLR engagement drives caspase-1 activation that cleaves pro-IL-1B which then gets secreted. Released IL-1B recruits immune cells to the site of infection/injury. Caspase-1 also cleaves Gasdermin-D (GSDM-D) that forms pores within the plasma membrane driving inflammatory cell death called pyroptosis. NLRP3 is the most extensively studied NLR. The NLRP3 gene is encoded by 9 exons, where exon 1 codes for pyrin domain, exon 3 codes for NACHT domain, and Leucine Rich Repeat (LRR) domain is coded by exon 4-9. Exon 2 codes for a highly disorganized loop that connects the rest of the protein to the pyrin domain and may be involved in NLRP3 regulation. The NLRP3 inflammasome is activated by many structurally divergent agonists of microbial, environmental, and host origin. Activated NLRP3 interacts with an adaptor protein, ASC, that bridges it to pro-Caspase-1 forming a multi-protein complex called inflammasome. Dysregulation of NLRP3 inflammasome activity is a hallmark of pathogenesis in several human diseases, indicating its highly significant clinical relevance. In this review, we summarize the existing knowledge about the mechanism of activation of NLRP3 and its regulation during activation by infectious and sterile triggers.

## Introduction

**Figure 1:**
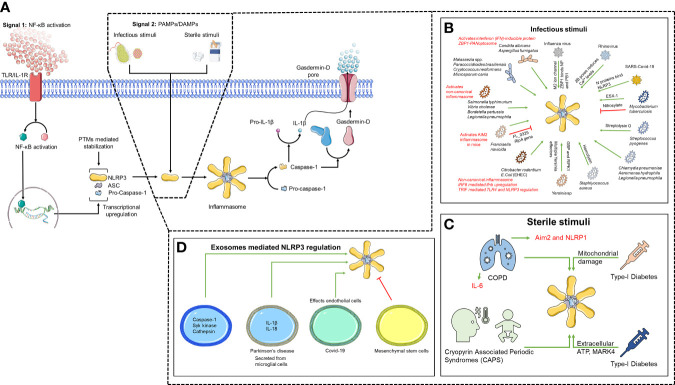
Two-step mechanism of NLRP3 activation. **(A)** TLR/IL1-R engagement leads to NF-κB activation driving transcriptional upregulation of inflammasome components (Signal 1). NLRP3 is then activated upon sensing unknown direct agonists (Signal 2), which can be **(B)** infectious, **(C)** sterile and can also be mediated through **(D)** exosomes.

Nod-Like Receptor (NLR) is the largest family of Pathogen Recognition Receptors (PRRs) that patrols the cytosolic environment. NLR engagement drives caspase-1 activation that cleaves pro-IL-1β which then gets secreted (1). Released IL-1β recruits immune cells to the site of infection/injury (2–8). Caspase-1 also cleaves Gasdermin-D (GSDM-D) that forms pores within the plasma membrane driving inflammatory cell death called pyroptosis (9–12). NLRP3 is the most extensively studied NLR (10–17). The *NLRP3* gene is encoded by 9 exons, where exon 1 codes for pyrin domain, exon 3 codes for NACHT domain, and Leucine Rich Repeat (LRR) domain is coded by exon 4-9. Exon 2 codes for a highly disorganized loop that connects the rest of the protein to the pyrin domain and may be involved in NLRP3 regulation (18). The NLRP3 inflammasome is activated by many structurally divergent agonists of microbial, environmental, and host origin (11, 19). Activated NLRP3 interacts with an adaptor protein, ASC, that bridges it to pro-Caspase-1 forming a multi-protein complex called inflammasome (2–8). Dysregulation of NLRP3 inflammasome activity is a hallmark of pathogenesis in several human diseases (20–24), indicating its highly significant clinical relevance. In this review, we summarize the existing knowledge about the mechanism of activation of NLRP3 and its regulation during activation by infectious and sterile triggers (**Figure 1**).

## Activation of NLRP3 Inflammasome: Two Signal Model

NLRP3 inflammasome formation follows a two-signal process (25). The engagement of TLR or cytokine receptors resulting in activation of NF-κB is called the priming (signal 1). NF-κB activation induces the transcription of NLRP3, ASC, pro-caspase-1 and pro-IL-1β regulating their cellular levels allowing NLRP3 activation specifically during pathogen invasion and endogenous threats. Apart from transcriptional regulation, NLRP3 is also stabilized by priming-induced post-translation modifications (PTM) (25, 26).

The molecular mechanism behind NLRP3 activation (signal 2) is believed to be the result of various cellular events involving K^+^ efflux, ROS generation, and release of cathepsin B due to phagosome rupture (10, 11, 19). It was believed that agonists are sensed by LRRs of NLRP3 inducing conformational changes leading to recruitment of inflammasome components (11),although, recent studies suggest that LRRs are dispensable for NLRP3 activation (18, 27). Given the variety of agonists, it is believed that NLRP3 actually senses an upstream unifying factor, e.g. cellular stress, commonly induced by all NLRP3 agonists (19, 25, 28). ROS was once believed to be a common upstream factor until recently where it was shown that ROS affects priming and not activation (29, 30). K^+^ efflux is one of the most accepted models of NLRP3 inflammasome activation (19, 25). NLRP3 agonists, nigericin and ATP, activates NLRP3 through K^+^ efflux. Nigericin is a K^+^/H^+^ ionophore while ATP-dependent NLRP3 activation engages P2X purinoceptor 7 receptor (P2X_7_R), a ligand-gated ion channel capable of K^+^ efflux (31–33). It was recently discovered that P2X_7_R couples with Two-pore domain Weak Inwardly rectifying K^+^ channel 2 (TWIK2) for K^+^ efflux, whereas P2X_7_R is responsible for influx of Na^+^ and Ca^2+^ (34, 35). P2X_7_R pores are believed to facilitate PAMPs/DAMPs entry to the cell (19, 36). However, some agonists of NLRP3 like monosodium urate crystals (MSU) and particulate asbestos are too large to be translocated through these pores (19). Moreover, several studies have shown K^+^ efflux-independent activation of NLRP3, demanding reexamination of the K^+^ efflux model (29, 37, 38). The lysosomal rupture model considers the size of agonists. Phagosome destabilization and rupture is caused by inefficient clearance of particulate agonists. The emanating release of Cathepsin B is believed to activate NLRP3 either directly or indirectly (11, 15, 16, 25). While several studies show cathepsin B inhibitor blocks lysosomal disruption and impair NLRP3 inflammasome (39), IL-1β processing in Cathepsin B deficient mice is comparable to that of wild-type mice (40, 41). Thus, cathepsin B inhibitors likely impair NLRP3 inflammasome activation by off-target effects. Lysosomal rupture also causes K^+^ efflux suggesting involvement of multiple pathways in activating NLRP3 (42–45). As most of NLRP3 agonists induces reactive oxygen species (ROS) and various ROS scavengers also impairs inflammasome activation (46, 47) suggesting that NLRP3 senses cellular stress (10, 11, 13, 15, 19, 25). NLRP3 activation requires interaction with Thioredoxin (TX) Interacting Protein (TXNIP) (48, 49), but, these finding could not be confirmed by others (50). Further, ROS activation also drives K^+^ efflux further suggesting that NLRP3 can be activated by multiple independent pathways. Moreover, the mitotic kinase, NEK7, facilitates NLRP3 activation and inflammasome assembly following activation by ATP and nigericin (30, 51, 52). NEK7 binds to LRR, hinge domain 2 (HD2) and the NACHT domain (53). Oridonin, the major active ingredient of the traditional Chinese medicinal herb *Rabdosia rubescens*, blocks NLRP3-NEK7 interaction by covalently modifying cysteine (C) 279 (54) on the interaction surface of NEK7 Moreover, NEK7 interacts with minimally active NLRP3 truncated mutant (NLRP3 1-686). NEK7 also interacts with NLRP3 1-665 which cannot be activated by various agonists (27). Thus, NEK7 interaction is not the primary requirement for NLRP3 activation, suggesting the possible involvement of other cellular factors. Lastly, various NLRP3 agonists cause mitochondrial dysfunction and release of mitochondrial ROS and mitochondrial DNA that activates NLRP3 (55–57). However, the direct or indirect nature of this interaction is yet to be investigated. Thus, it is likely that either a common upstream factor activates, or multiple pathways lead to inflammasome activation. It is also likely that a combination of factors leads to activation of NLRP3. Since, inflammasome formation follows prion-like polymerization leading to Supramolecular Organization Centers (SMOCs) it is also possible that some threshold effect is required to activate NLRP3 which may be achieved by involving multiple pathways. Such a mechanism would also account for rapid NLRP3 activation by multiple agonists.

## NLRP3 Regulation

### Protein Binding

Since NLRP3 activation mechanism remains elusive, it is plausible that a common upstream factor activates/regulates NLRP3. Most of the proteins interacting with NLRP3 binds to the NACHT-LRR domain suggesting the importance of these domains in NLRP3 regulation. Heat shock protein (Hsp) 90 chaperone complex binds to NLRP3-LRR domain (58), however, it is unclear whether the chaperone complex facilitates proper folding or unfolding of LRRs after stimulation. Further, redox sensitive TXNIP interacts with NACHT-LRR domain of NLRP3 and knocking down TXNIP also reduces NLRP3 inflammasome response (49). Interestingly, TXNIP^-/-^ BMDMs showed no effect on inflammasome response (50). Mitotic kinase, NEK7, binding is shown critical for NLRP3 inflammasome response (30, 52, 53). Additionally, E3 ubiquitin ligase tripartite motif-containing protein 31 (TRIM31) promotes proteasomal degradation of NLRP3 thus attenuating NLRP3 inflammasome response (59). NLRP3 inflammasome response is impaired by binding of Pyrin only proteins (POPs) and CARD only proteins (COPs). POPs and COPs are reviewed in detail in Le H.T. et al., 2013 (60). Interestingly, a few interacting partners binds to PYD domain suggesting a possible involvement of PYD in NLRP3 inflammasome response. Mitochondrial anti-viral signaling protein (MAVS) interaction with NLRP3 PYD is critical for mitochondrial localization and NLRP3 inflammasome response (61). Further, microtubule-affinity regulating kinase 4 (MARK4) also interacts with the PYD domain of NLRP3 which is critical for positioning and translocation of NLRP3 to form the inflammasome (62). Although several binding partners of NLRP3 are discovered, the mechanism behind their action is not yet understood. One of the caveats with these studies are the use of special agonists and the importance of these protein-protein interactions is specific to the respective agonists. It is important to evaluate the role of these proteins under different conditions.

### Post-Translational Modifications

Various PTM regulate innate immune signaling through different cellular processes [Reviewed in (63)]. NLRP3 expression is regulated by both transcriptional and post-translational modifications (PTM) (26). NLRP3 is phosphorylated by Spleen tyrosine kinase (Syk) (64–67), Death-associated protein kinase (DAPK) (68), Transforming growth factor beta-activated kinase 1 (TAK1) (69) and Extracellular Signal-Regulated Kinase 1 (ERK1) (70) in infection models. It is unclear whether these kinases specifically phosphorylate NLRP3 or other inflammasome components. Dephosphorylation of NLRP3 at serine 5 by Protein phosphatase 2 (PP2A) (71), and tyrosine 859 by protein tyrosine phosphatase non-receptor type (PTPN22) primes NLRP3 for activation (72, 73). Interestingly, phosphorylation at serine 295 by Protein kinase D (PKD) activates NLRP3, whereas, by Protein kinase A (PKA) abrogates NLRP3 inflammasome activation (22, 72–74). It is unclear how the same PTM at the same site leads to two different outcomes. It is likely that a combination of PTMs rather than a single PTM regulate NLRP3 inflammasome. PYD, NACHT and LRRs are ubiquitinylated by TRIM31 (59, 75), Ariadne homolog 2 (ARIH2) (76), and membrane-associated RING finger protein 7 (MARCH7) (77), respectively promoting proteasomal degradation of NLRP3. Finally, deubiquitylation of LRRs by BRCA1/BRCA2-containing complex subunit 3 (BRCC3) is required for NLRP3 oligomerization and activation (78). In the resting state, mitochondrial E3 ubiquitin protein ligase 1 (MUL1) SUMOylates NLRP3 at multiple sites. Following activation, sentrin-specific protease 6 (SENP6) and 7 (SENP7) deSUMOylates NLRP3 promoting inflammasome function (79). However, further studies are required to confirm the role of SUMOylation in NLRP3 activation. Nitrosylation is associated with NLRP3 inhibition (80–82). Mao et al. demonstrated that treatment with nitric oxide donor, SNAP, inhibits inflammasome function in mouse peritoneal macrophages, THP1 cells and human peripheral blood mononuclear cells (PBMCs) (81). During mycobacterial infection of mice, NLRP3 is nitrosylated by IFN-γ induced nitric oxide synthase (iNOS) (82). NO-mediated inhibition is specific for NLRP3 as the AIM2 and NLRC4 inflammasomes are only moderately affected (80). NO-mediated inhibition operates through thiol modifications of cysteine residues. Further, a recent study has shown cysteine dependent NLRP3 inflammasome response to sterile agonists whereas response to Fransicella novicida U112 was cysteine independent (18). Cysteine thiol groups are strongly nucleophilic, and the availability of d-orbital electrons help attain multiple oxidation states (83–86). Such chemistry provides versatility of forming a molecular code that can efficiently respond to oxidative stress (87). Depending on the cell redox state, cysteines can undergo various reversible and irreversible modifications (83–86). While reversible modification of cysteine functions as a signaling intermediate, irreversible intermediates are rarely involved in signaling. NLRP3 has 43 cysteines and is regulated by nitrosylation (82), however, which cysteines are modified is still unknown. PTMs are very strong regulator of protein functions. Whether a single PTM regulate NLRP3 inflammasome or multiple PTMs works in tandem to regulate NLRP3 is yet to be determined. Further studies evaluating the PTMs on NLRP3 following stimulation by different agonists are required to fully understand the role of PTMs on NLRP3 activation.

## NLRP3 and Its Association With Diseases

### Bacterial Infection

Several studies have revealed a crucial role of the NLRP3 inflammasome in bacterial infections (1). The Pathogen associated molecular patterns of Gram-negative bacterial pathogens like *Yersinia* spp, *Francisella* spp and *Salmonella* spp are recognized by more than one PRR (2). However, NLRP3 activation is a common response to all 3 pathogens. *Francisella novicida* U112 activates human NLRP3, whereas in mice, AIM2 is the predominant inflammasome that responds to *Francisella* (Fn) infection (3), and a recent study shows this response is debilitating to the host (4). However, NLRP3 deficient mice display improved survival in Fn infection indicating the presence of a contrasting non-inflammasome role of NLRP3 (5). NLRP3 share structural similarity to CIITA which makes it likely to function as a transcriptional regulator as well. A non-inflammasome function of NLRP3 is interesting and requires further investigation. However, it is still perplexing why a single protein belonging to such a big super-family have multiple functions. *Salmonella enterica* serovar Typhimurium activates the NLRC4 and NLRP3 inflammasomes in macrophages and both are required for efficient IL1β release (6). Given the critical role that NLRP3 activation plays in mounting an effective immune response to bacterial infection, it is not surprising that bacterial pathogens have developed tools to subvert inflammasome activation (7). Majority of the evidence of inflammasome activation in *Yersinia* infections have been discovered while identifying virulence factors that disabled the host immune response. *Yersinia* effectors YopK, YopB, YopD, YopM and YopJ have all been implicated to regulate the NLRP3 inflammasome through different mechanisms (8–11). In case of Fn, the FL_0325 virulence factor and gene *ripA* were discovered to suppress NLRP3 and AIM2 activation and mutant strains lacking these genes generated a stronger innate immune response than wild type Fn (12–14). It is interesting that how effectors are targeted towards NLRP3 rather than blocking the end-product of inflammasome activation, i.e., caspase-1. This suggests that NLRP3 might have functions beyond caspase-1 activation. Among other Gram-negative pathogens known to activate the NLRP3 inflammasome are *Aeromonas hydrophila, Bordetella pertussis, Vibrio cholerae* and *Legionella pneumophila* (15–21). *V. cholerae* and Endotoxin*B. pertussis* activate more than one type of inflammasome including NLRP3 *via* canonical and non-canonical pathways (22, 23). Several host factors are also involved in the activation of the NLRP3 inflammasome during bacterial infections. For example, IRF8 promotes *Ifnb* transcription which in turn activates caspase-11 to trigger the NLRP3 inflammasome in murine macrophages infected with *Citrobacter rodentium* (24). In another study, TRIF was identified as an important bridge between TLR4 and NLRP3 in enterohemorrhagic *Escherichia coli* (EHEC) and *C. rodentium* infected cells (25). Noncanonical activation of NLRP3 was found to be caspase-4 dependent in macrophages infected by *L. pneumophila, Y. pseudotuberculosis* and *S. Typhimurium* (26). Further, in *Yersinia* infected cells, RIPK-1 and Guanylate binding proteins are critical regulators of pyroptosis or apoptosis (27, 28).

Examples of NLRP3 inflammasome response to virulence factors of Gram-positive bacterial pathogens include hemolysins secreted by *Staphlyococcus aureus* (*Sa*) which activates NLRP3 and induces IL-1β secretion (88, 89). However, another group showed that staphylococcal hemolysins are dispensable for NLRP3 activation (88, 89). *Sa*-associated PAMPs also cooperate with hemolysin to activate NLRP3 (90). Similarly, streptolysin O released from *Streptococcus pyogenes* is important for NLRP3 activation (91, 92). NLRP3-dependent IL-1β is also crucial for protection against *Chlamydia pneumoniae* infection (93). It is interesting to see how NLRP3 activation by gram-positive bacteria broadly use K^+^ efflux, whereas for Gram-negative bacteria the mechanism is more varied. *Mycobacterium tuberculosis* (Mtb) inhibits the inflammasome response activation by nitrosylating NLRP3 (82). ESX-I secretion system of both Mtb and *Mycobacterium marinum* activates NLRP3 activation (94–96). Some bacteria, like *Listeria monocytogenes* induce multiple inflammasomes, including NLRP3, Aim-2 and NLRC4, and trigger inflammasome formation by mixed NLRs (97–102). These observations highlight the importance of NLRP3 in detection of various bacteria and the importance of the inflammasome response during bacterial infections.

### Fungal Infection

Many fungal species have been shown to activate the NLRP3 inflammasome that include *C. albicans, A. fumigatus, Malassezia spp., Paracoccidioides brasiliensis, Cryptococcus neoformans* and *Microsporum canis* (103). NLRP3 deficient mice are susceptible to *C. albicans* infection (104). Further, hypheal forms of fungi are more potent inducers of NLRP3 inflammasome than yeast which activates NLRP3 with candidalysin (105, 106). *C. albicans* and *A. fumigatus* also activates the interferon (IFN)-inducible protein ZBP1-PANoptosome, resulting in NLRP3 inflammasome activation and PANoptosis (107). In *A. fumigatus*, the activation of NLRP3 inflammasome is dependent on ROS production and K^+^ efflux (108). Syk kinase activity downstream of Card9 activation by the fungal sensor Dectin1, is required for NLRP3 activation (64, 109), however, the precise mechanism of NLRP3 activation by fungal PAMPs is unclear, contributing to the bottleneck in development of new drug targets to treat fungal infections. NLRP3 activation through fungal activation require further studies to elaborate the mechanism of activation.

### Viral Infection

NLRP3 is the only member of the NLR family that plays a role detecting viral RNA and proteins in viral infections. Both DNA and RNA viruses have been shown to activate the NLRP3 inflammasome with Sendai and Influenza virus being the first viruses discovered to do so (110). The interferon-inducible protein, thus activating the NLRP3 inflammasome (111). The cytosolic dsRNA sensor DHX33 which is a member of DExD/H-box helicase family, can interact with NLRP3, activating it (112, 113). Vivoporins are virus-encoded proteins with ion channel activity that cause changes in membrane stability which can be recognized by NLRP3 and activate the inflammasome (114). NLRP3 activation by influenza virus is proposed to be driven by the viral M2 ion channel that transports H+ ions out of trans-Golgi network but also leads to activation of other ion channels that drive K+ efflux (115, 116). In Rhinovirus, the 2B protein activates NLRP3 by creating pores and reducing the level of Ca^2+^ in ER and Golgi membranes (117). Inhibition of NLRP3 activation during Respiratory syncytial virus (RSV) infection decreases lung pathology *in vivo* (118). Recent study shows the SARS-CoV-2 N protein induces proinflammatory cytokines by promoting the assembly of NLRP3 inflammasome *via* direct interaction with NLRP3. Using a mouse model of infection, the authors have demonstrated that activation of NLRP3 by N protein has been associated with lung injury and a cytokine storm, the two hallmarks of Covid-19 infection (119, 120).

### Cryopyrin Associated Periodic Syndrome

Cryopyrin Associated Periodic Syndromes (CAPS) are a spectrum of chronic inflammatory diseases caused by gain of function mutations in the NACHT and LRR domain of NLRP3 (20, 121–123). These diseases include Familial cold autoinflammatory syndrome (FCAS), Muckle-Wells Syndrome (MWS) and Neonatal onset multi-systemic inflammatory disease (NOMID)/Chronic infantile neurological cutaneous articular syndrome (CINCA). Macrophages and monocytes isolated from CAPS patients spontaneously secrete IL-1β in the absence of any inflammatory stimuli (124). While recurrent fever and joint-pains are clinical symptoms of FCAS and MWS (125), NOMID patients present severe neurological and developmental complications (125, 126).

### Chronic Obstructive Pulmonary Disease

COPD presents as a combination of emphysema, chronic bronchitis, and chronic airway obstruction (127, 128). While inflammation and IL1β release is central to progression of COPD, there are mixed reports of whether and which inflammasome activation causes severe damage to the lungs (129–131). Exposure to cigarette smoke and other particulate matter is a major cause of COPD and its role in activating the NLRP3 inflammasome is well documented (132, 133). Further, evidence of NLRP3 activation in stable and exacerbated COPD was found in sputum and plasma samples of patients, in an *in vitro* model of COPD and in patients with neutrophilic asthma (134–136). In contrast, one study found no induction of NLRP3 but an increase in inflammatory cytokines like IL-6 in the broncho alveolar lavage (BAL) fluid of patients with stable COPD (137). Another genetic study of polymorphisms in COPD patients, identified a single nucleotide polymorphism in NLRP1 which correlated with decreased lung function (138). Additionally, other studies discovered the activation of the AIM2 inflammasome consistent with IL1β release in BAL, lung tissue and peripheral blood mononuclear cells isolated from COPD patients (139, 140). Taken together, there is ample evidence of IL-1 cytokines and inflammasome activation in stable and exacerbated COPD but whether NLRP3 activation drives disease progression and the mechanism behind it is yet to be elucidated.

### Diabetes

Type I (T1D) and Type II (T2D) diabetes differ in the mechanism by which insulin resistance develops (141), but the role of inflammasomes have been implicated in both. In a study from Brazil, 2 SNPs in the NLRP3 gene were found in pediatric patients with T1D (142). In a murine model of T1D, mitochondrial DNA from diabetic mice displayed the ability to induce IL1β which could be inhibited in NLRP3 ^-/-^ macrophages (143). Further, NLRP3 deficient mice were unable to develop T1D (144). Although the exact mechanism of how the NLRP3 inflammasome contributes to T1D is yet to be discovered, the existing data indicates a crucial role for the former. As for T2D, studies have been more illuminating. IL1β release and the activation of NLRP3 are central to T2D (21, 145, 146). Monocyte-derived macrophages from T2D patients displayed high NLRP3, IL-18 expression, caspase-1 cleavage and NLRP3 dependent IL1β secretion (147). High extracellular ATP in T2D results in the activation of the P2X7 receptor that in turn activates the NLRP3 inflammasome (148). Additionally, activation of NLRP3 can be controlled by the microtubule affinity regulating kinase (MARK4) which in turn is regulated by the E-74 like ETS transcription factor (ELF3). High glucose increased ELF3 expression in HUVECs and triggered the NLRP3 inflammasome (149). Diabetic markers such as saturated fatty acids and islet amyloid polypeptide also activate NLRP3 (50, 150). Recent clinical trials have shown potential in IL-1β blocking therapies further establishing an important role of IL-1β in progression of diabetes (151). However, treatment with Canakinumab, an IL1β inhibitor, did not reduce the risk of diabetes in patients with pre-diabetes (152).

## Role of Exosomes in NLRP3 Inflammasome Activation

Extracellular vesicles (EVs) are membrane enclosed nano-bodies (size 30-1000nm) that facilitate cargo transport and signal transduction regulating physiological and pathological processes (153). Numerous studies have established that exosomes can critically influence the progression of inflammatory diseases by modulating the NLRP3 inflammasome (154–159).

Inflammasome-associated EV facilitate inflammatory responses in neighboring and distantly located recipient cells (159, 160). Particulate NLRP3 activators, like calcium oxalate, monosodium urate and β-glucan can activate EV-mediated cargo release in human macrophages (154). EV cargo contains Caspase-1, Syk kinase and Cathepsin indicative of NLRP3 inflammasome associated EV release upon lysosomal damage (161). NLRP3 inflammasome derived exosomes promotes inflammation by transducing signal to neighboring cells (162). NLRP3 activation causes exosome release, carrying IL-1β and IL-18, from microglial cell membrane promoting neuro-inflammation in Parkinson’s disease (162). Latest reports show that exosomes isolated from a severe COVID-19 patient’s plasma can exert its effect on human endothelial cells and liver endothelial cells increasing the expression of NLRP3, IL-1β and caspase-1 mRNA (160). Interestingly, EVs can also have inhibitory effect on NLRP3 activation. Mesenchymal stem cells derived exosomes from umbilical cords can attenuate caspase-1 production, resulting in lower levels of IL-1β and IL-18 thus inhibiting NLRP3 activation (163). Stem cell-derived exosomes can repair ischemic muscle injury by inhibiting the Rb1-mediated NLRP3 inflammasome pathway highlighting anti-inflammatory potential of exosomes (164).

## Conclusion

In summary, NLRP3 activation by sterile and infectious agents display significant differences in mechanism. Moreover, the activation mechanism between gram-positive and gram-negative bacteria also differs greatly. Further, bacteria induced NLRP3 activation mechanism is different from NLRP3 activation by fungi and viruses. Such a difference in activation mechanism account for versatility of NLRP3 to react to different threats. Such differences may also be important in channel downstream signaling for launching adaptive immune response to the threat. In the two-decades of inflammasome research, scientists have discovered various mechanism of NLRP3 activation. While these discoveries have significantly advanced the inflammasome biology field, they have opened several new questions that will require further attention.

## Author Contributions

All authors listed have made a substantial, direct, and intellectual contribution to the work and approved it for publication.

## Conflict of Interest

Author AN was employed by Luminex Corporation.

The remaining authors declare that the research was conducted in the absence of any commercial or financial relationships that could be construed as a potential conflict of interest.

## Publisher’s Note

All claims expressed in this article are solely those of the authors and do not necessarily represent those of their affiliated organizations, or those of the publisher, the editors and the reviewers. Any product that may be evaluated in this article, or claim that may be made by its manufacturer, is not guaranteed or endorsed by the publisher.
